# Investigation on the Effects of the Formation of a Silver “Flower-Like Structure” on Graphene

**DOI:** 10.1186/s11671-016-1793-y

**Published:** 2017-01-18

**Authors:** Rozalina Zakaria, Siti Fatimah Az Zahra Yusoff, Kok Chung Law, Chin Seong Lim, Harith Ahmad

**Affiliations:** 1 Photonics Research Centre, University of Malaya, 50603 Kuala Lumpur, Malaysia; 2 Department of Mechanical, Materials and Manufacturing Engineering, The University of Nottingham Malaysia Campus, Jalan Broga, Semenyih, 43500 Selangor Darul Ehsan Malaysia

**Keywords:** Surface plasmon resonance, Silver nanoparticles, Electrochemical deposition technique, CST microwave studio

## Abstract

In this report, we experimentally investigate the formation of “flower-like silver structures” on graphene. Using an electrochemical deposition technique with deposition times of 2.5 and 5 min, agglomerations of silver nanoparticles (AgNPs) were deposited on the graphene surfaces, causing the formation of “flower-like structures” on the graphene substrate. Localized surface plasmon resonance (LSPR) was observed in the interaction between the structures and the graphene substrate. The morphology of the samples was observed using a field-emission scanning electron microscope (FESEM) and Raman spectroscopy. Thereafter, the potential of the flower-like Ag microstructures on graphene for use in Raman spectroscopic applications was examined. The signal showed a highest intensity value after a deposition time of 5 min, as portrayed by the intense local electromagnetic fields. For a better understanding, the *CST Microwave Studio* software, based on the finite element method (FEM), was applied to simulate the absorption characteristics of the structures on the graphene substrate. The absorption peak was redshifted due to the increment of the nanoparticle size.

## Background

Nanoplasmonics has been an intense subject in science and technology research fields that include the fabrication process and optical characterization of metal nanoparticles [1]. Surface plasmon resonance can be described as a quantum of electron charge density that oscillates at a metal-dielectric interface [2]. It has been reported that noble metal nanoparticles have unique electronic and optical properties. Particularly, plasmons on graphene are a new finding in nanoplasmonic applications, as they can confine a high amount of electromagnetic energy at subwavelength scales, thus leading to a strong surface plasmon resonance (SPR) at a metal-dielectric interface [3]. Moreover, because of the 2D characteristic of the collective excitations, the excitation of surface plasmons (SPs) in graphene becomes strongly restrained compared with those in normal noble metals [4]. Graphene thin films decorated with random distributions of metallic nanoparticles have been shown to enhance optical and electronic properties, which has been studied by Reza et al. [5] by modeling the influence of graphene substrate with gold nanoparticles with distance on the graphene sheet.

Graphene or 2D carbon sheets might be ideal nanoscale substrates to attain metal nanoparticle (NP) films [6]. Graphene itself has been recognized as an ideal optical material for optoelectronic applications replacing silicon and indium tin oxide because of the desirable properties offered, such as a good nonlinear effect, a low loss compared with usual metals [7], and a high carrier mobility. An effect of localized surface plasmon resonance (LSPR) exists due to the interaction between plasmonic nanoparticles, such as gold (Au) and silver (Ag), with the light wave on a surface of graphene enhancing the performance of nanoplasmonics, even though the use of Au and Ag is less efficient than copper (Cu) or nickel (Ni) [8]. A tunable surface plasmon on graphene can be achieved by varying the size, structure, and shape of the metal nanoparticles [9], thus resulting in a variation of the wavelength of light detected. In this research, silver (Ag) nanoparticles were chosen due to their unique characteristics, such as high detection accuracy [10] and ability to provide a sharp SPR dip [11], which leads to a device with good sensitivity. In addition, Ag nanoparticles exhibit the strongest interaction between plasmonics and light and a greater scattering cross section compared with other noble metals [12].

In this study, the manner of LSPR of Ag nanoparticles coated on graphene surfaces with two different deposition times and structures was investigated as a preliminary approach. An electrochemical technique was applied to deposit Ag nanoparticles on a graphene substrate. However, the mechanism of the formation of flower-like silver nanostructures by the electrochemical technique has not been fully explored. Two samples with Ag nanoparticles were obtained at deposition times of 2.5 and 5 min to observe the formation of Ag nanoparticle structures. The morphology of the nanoparticles was then observed using a field-effect scanning electron microscope (FESEM). The existence of graphene was proven by Raman spectroscopy analysis with the addition of integrating metallic nanoparticles. A surface profiler was used to check the thickness of the Ag nanoparticles deposited on the graphene surface. In addition, the LSPR effect can be clarified by analyzing the absorption spectrum, whereby CST Microwave Studio (MMS) was used to simulate the light absorption characteristics of these two samples to support the experimental result.

## Methods

### Fabrication of Ag Nanoparticles

**Fig. 1:**
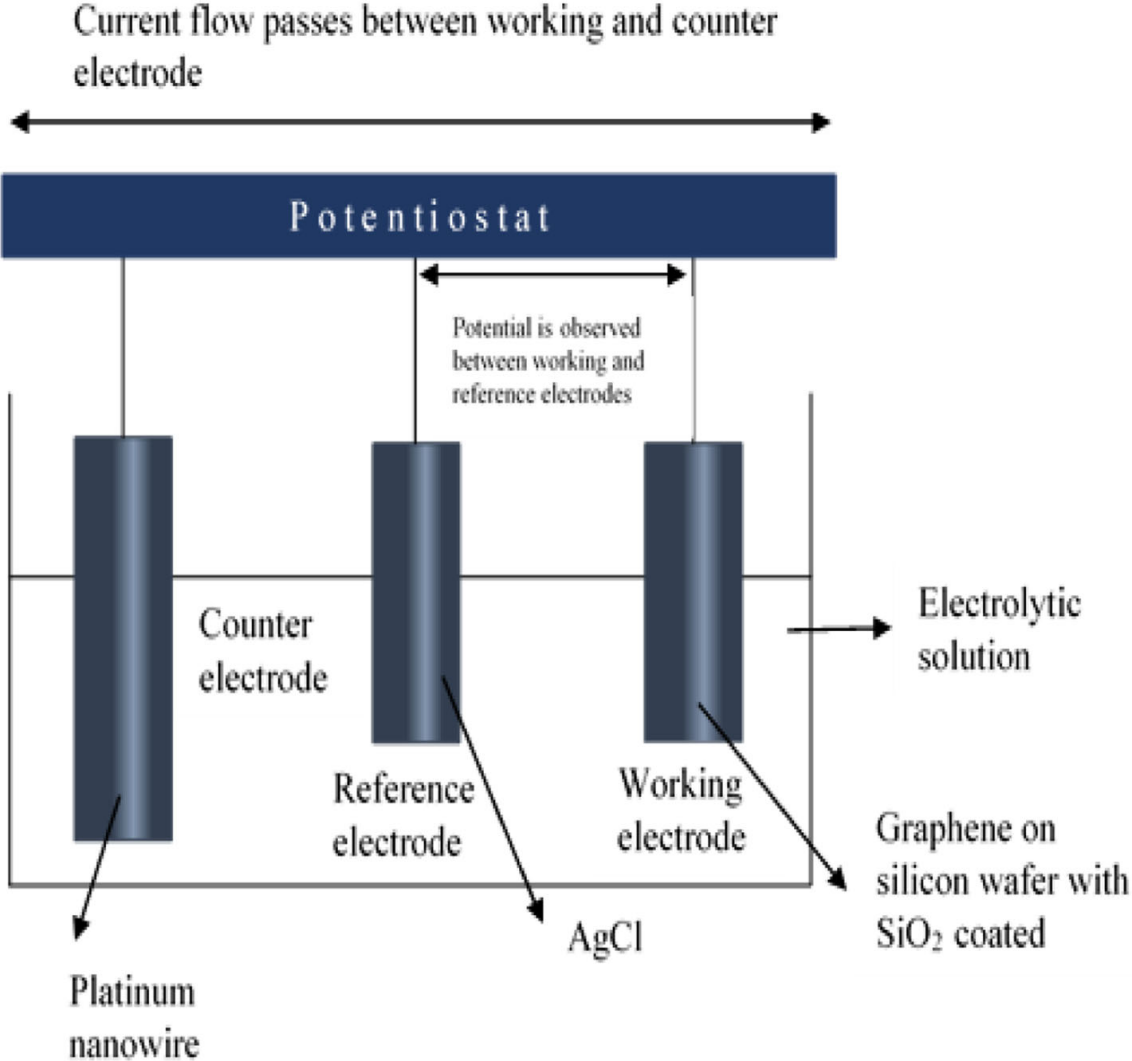
Schematic diagram of electrochemical deposition technique

The electrochemical deposition of Ag nanoparticles was conducted using a three-electrode electrochemical cell immersed in a silver ammonia ([Ag(NH_3_)_2_]OH) solution. First, 425 mg of AgNO_3_ was added to 50 mL distilled water to produce 50 mL of 50 mM AgNO_3_. The silver ammonia solution was obtained by mixing dropwise ammonia (1 wt%) with 10 mL of the AgNO_3_ solution (50 mM). The mixture was then stirred several times until the color of the solution changed to a light color, thus resulting in the formation of [Ag(NH_3_)_2_]OH with a concentration of 40 mM. There are two methods of electrochemical deposition—cyclic voltammetry (CV) and chronoamperometry (CA) electrodeposition—and these two processes were carried out in the prepared solutions using a potentiostat/galvanostat (Versastat 3 Applied Research Princeton, USA). The three-electrode system is described in Table 1 (below), and the schematic diagram of the system is shown in Fig. 1.

**Table 1:** Three-electrode system used in the electrochemical deposition technique

Materials/substrate	Function
Graphene on silicon wafer coated with SiO_2_	Working electrode
Platinum nanowire	Counter (auxiliary) electrode
AgCl	Reference electrode

### Characterization of Ag Nanoparticles

For the characterization of Ag nanoparticles on graphene, a Quanta 400F scanning electron microscope (FESEM) was used to obtain high-resolution images of the structures down to the nanoscale. A KLA-TENCOR P6 surface profiler was then used to measure the thickness of the Ag nanoparticles (Table 2), and a Renishaw InVia Raman spectroscope was used to measure the Raman spectra of the samples. Finally, CST Microwave Studio simulation software was used to simulate the absorption characteristics of the drawn samples.

**Table 2:** Measurement of the height of the Ag nanoparticles deposited on the graphene surface using the surface profiler

Height of Ag nanoparticles from graphene surfaces (nm)	Samples	
	2.5 min	5.0 min
1st reading	326.45	515.54
2nd reading	156.98	416.01
3rd reading	172.14	403.21
4th reading	216.12	443.44
5th reading	302.52	335.68
Average	234.84	422.78

## Results and Discussion

### Electrodeposition of Ag Nanoparticle at 2.5 and 5 min

Cyclic voltammetry (CV) was implemented to determine the suitable potentials to be used in the experiment. The potentiostat was applied onto a working electrode linearly back and forth to produce a voltammogram (also known as a scan) [13]. After a few cycles, the optimum potential that could be used was 0.6 V, and the Ag nanoparticles were deposited on the graphene surface using the chronoamperometry (CA) method with two deposition time sets: 2.5 and 5 min.

**Fig. 2:**
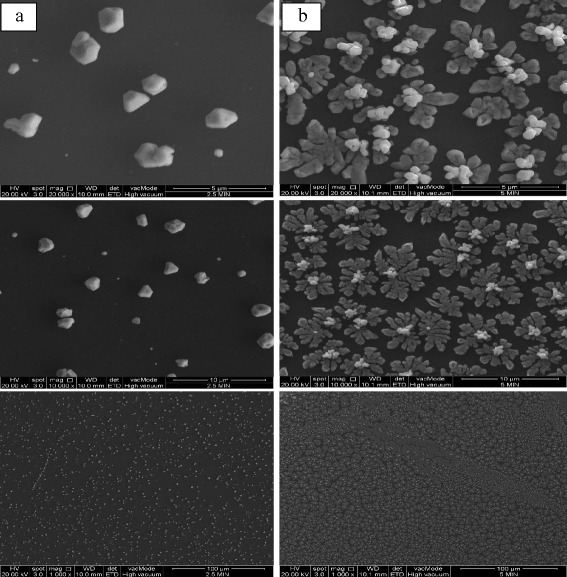
SEM image (magnification of ×20,000, ×10,000, and ×1000) of graphene surfaces coated with silver (Ag) nanoparticles with deposition time of **a** 2.5 min, **b** 5 min using CA method

Figure 2 shows the SEM images of the graphene surfaces coated with Ag nanoparticles for deposition times of 2.5 and 5 min. In the electrodeposition process, electrons flow from a platinum nanowire (counter electrode) towards the graphene surfaces (working electrode), as shown in Fig. 1. Ag cations that move freely inside the electrolyte solution gain electrons on the graphene surfaces and become stable Ag atoms, which is shown in the proposed reaction below:

$$ \mathrm{C}+2\left[\mathrm{A}\mathrm{g}{\left({\mathrm{NH}}_3\right)}_2\right]\mathrm{O}\mathrm{H}\to \left[\mathrm{C}{\left({\mathrm{NH}}_3\right)}_4\right]{\left(\mathrm{O}\mathrm{H}\right)}_2+2\mathrm{A}\mathrm{g} $$

Ag atoms moved and deposited randomly on the graphene surfaces, thus, becoming Ag nanoparticles. In Fig. 2a, Ag nanoparticles start to form but in small sizes (nanometer and micrometer scales). When the deposition time became longer (5 min), more Ag cations were reduced to Ag atoms. They preferred to move towards the area with a high deposition of Ag nanoparticles and formed a micro-flower nanoparticle structure, as shown in Fig. 2b. These flower-like Ag microstructures, composed of different intertwined plates, produced a hierarchy, and created a structure with abundant interstitial sites [14] after a period of deposition. The Ag micro-flower area (which is large in size) increased the conductivity, and this resulted in the agglomeration of more electrons in the areas with several Ag microstructures, exhibiting flower-like morphologies and tending to assemble together. Moreover, it was also observed that more Ag micro-flower structures were formed as more Ag nanoparticles stacked together, with several layers accumulating when the deposition time increased. The general morphology development of these formations is that the current initially increases with time, representing the nucleation and growth of the NPs that are (largely) diffusionally isolated, reaching a peak value followed by a decrease with time due to the diffusional overlap (and planar diffusion) of Ag^±^ to the resulting NP array [15]. This is worthy of further study to chemically understand the mechanism process.

### Raman Spectroscopy

**Fig. 3:**
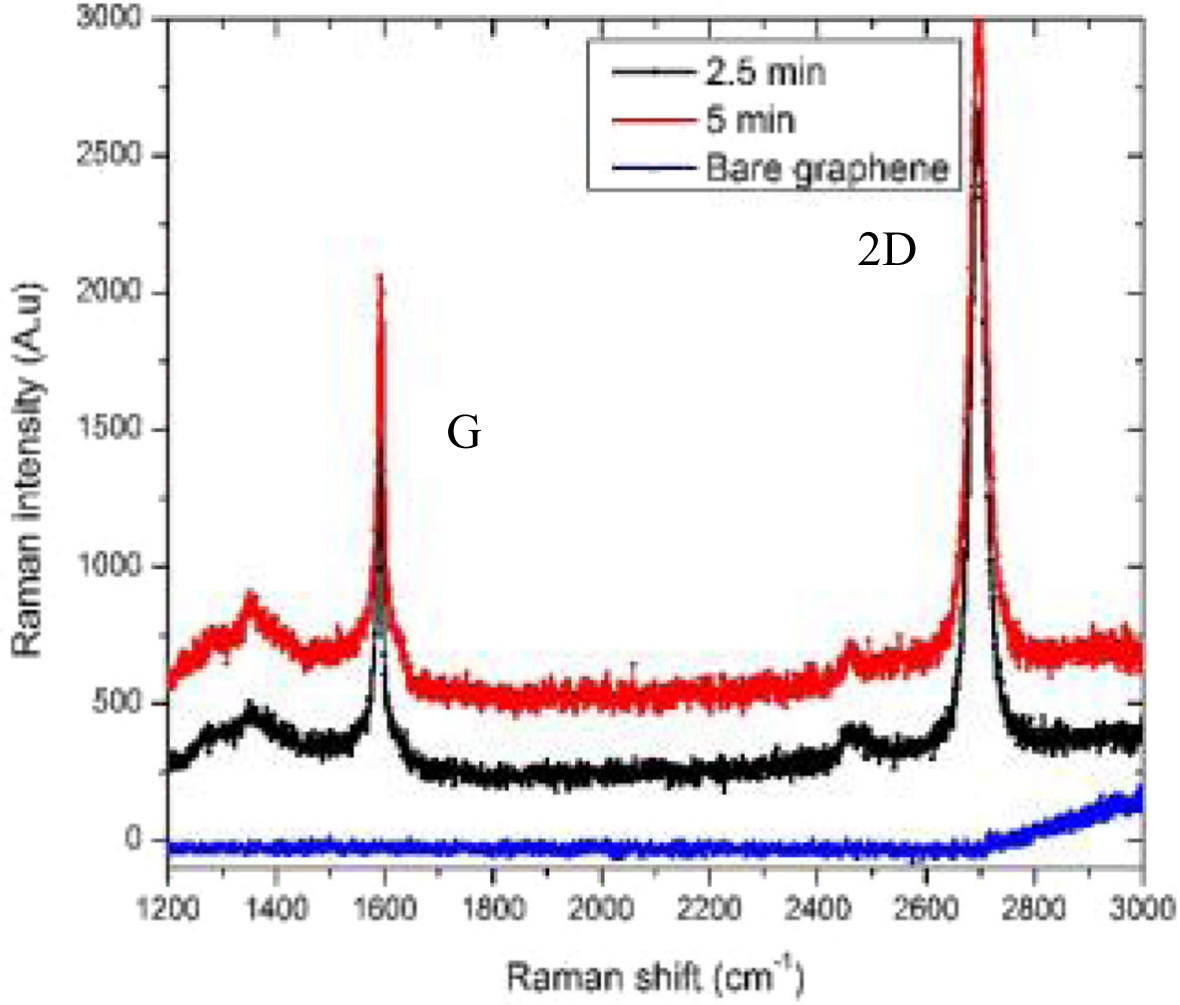
Raman spectra of the samples with deposition times of 2.5 and 5 min, measured at 514 nm

Raman spectroscopy was used to characterize the samples and study the localized surface plasmon resonance (LSPR) of the Ag nanoparticles deposited on the graphene surfaces. Figure 3 shows the Raman spectra of the two samples. The Raman spectra have two obvious peaks, which were approximately located at 1600 and 2700 cm^−1^ and corresponded to the G- and 2D-bands of graphene [16]. The G-band arises due to the small domains of graphite that result in the vibration of sp^2^-bonded carbon atoms that belong to the E_2g_ irreducible, while the 2D-band comes from the second-order process of two phonons [17]. Raman spectroscopy was performed with a Renishaw single monochromator equipped with a 514-nm excitation energy source. The Raman spectrum consists of several distinct peaks characterized by their position width, height, and area [18]. A useful notation is *I* for peak height, so here, *I*(G) is the height of the G peak, as shown, and the *I*(G)/*I*(2D) peak ratios are 0.6 and 0.7 for 2.5 and 5 min, respectively. Several measurements were performed at the same spot and at different spots on the same sample. A sample with a structure size substantially larger than the laser spot size (~1.5 μm^2^) is preferable to avoid edge effects [18]. The fitting of a powerful microscope to the Raman spectrometer enables the analysis of micrometer-sized particles of material. When only univariate Raman models are used, the speed of Raman spectral imaging can be significantly increased by wide-field Raman microscopy; the laser is expanded to illuminate the entire area of the sample, and the scattered Raman light corresponding to selected wavenumbers are imaged on a CCD. In comparison with bare graphene, after the deposition of Ag nanoparticles occurred under the 2.5 and 5 min conditions, the intensity of G and 2D increased drastically due to the strong interaction between the surface of the Ag nanoparticles on the graphene and the excitation light. Moreover, the LSPR effect of the Ag nanoparticles was higher at the 5-min deposition time, as the size of the Ag nanoparticles increased and the distance between the nanoparticles decreased, as shown in Fig. 2b, resulting in a stronger coupling between the nanoparticles and the small area of the exposed graphene surface. It can be proven that when more nanoparticles are deposited on the graphene surface, more excitation will occur and the Raman intensity will become stronger [19]. However, after some time, the intensity will start to decrease due to the excessive agglomeration of Ag nanoparticles deposited on the graphene surface [16]. This shows that this work can be reported as a sensitive SERS substrate for folic acid detection using graphene oxide/Ag nanoparticle hybrids [20]. Ag nanoparticles have a strong UV-vis absorption and local surface plasmon resonance effect and have a brilliant future in both SERS and nonlinear optics.

### CST Computer Simulation

**Fig. 4:**
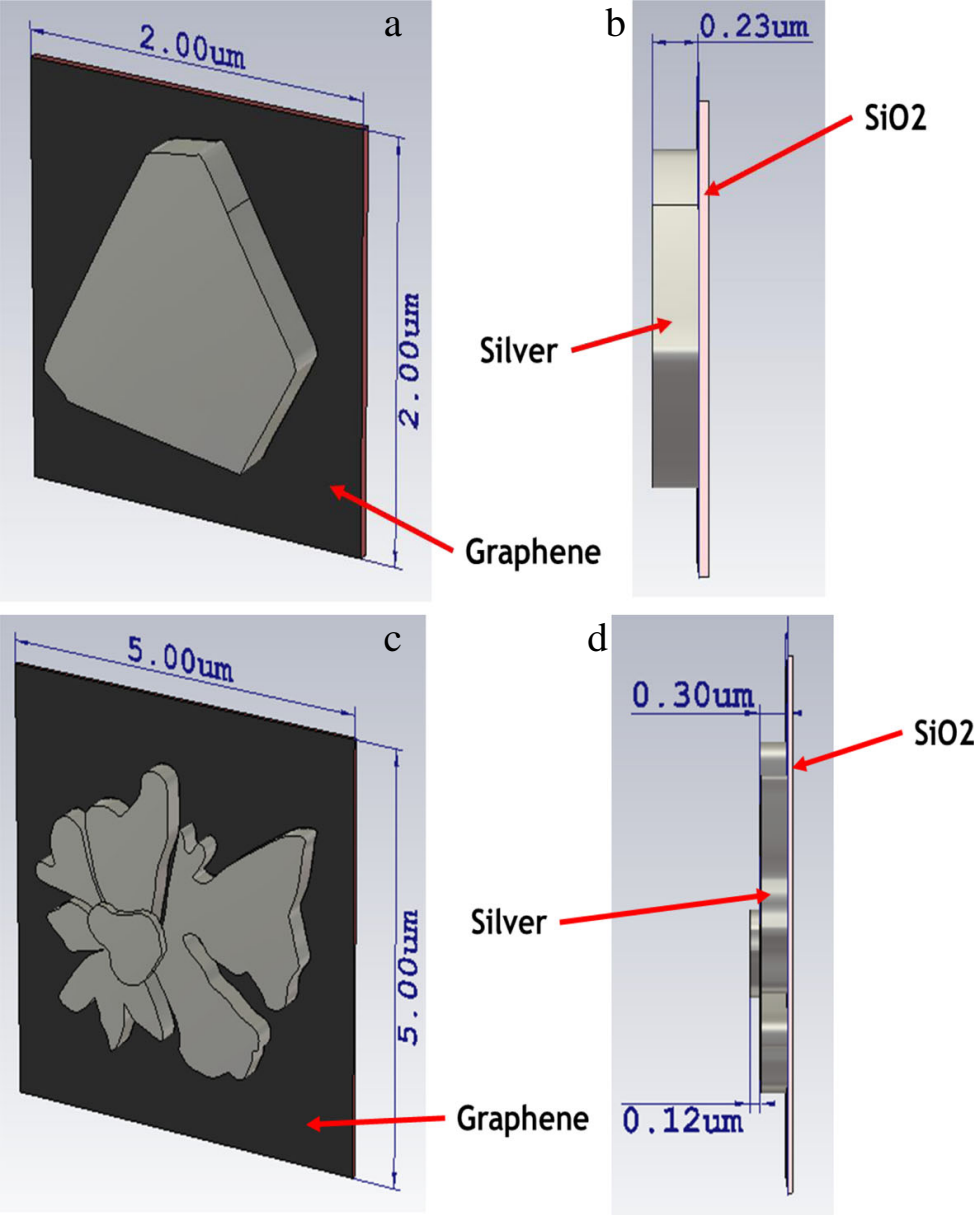
Small-size Ag nanoparticles, **a** front view and **b** side view; Ag micro-flower nanoparticles, **c** front view and **d** side view

**Fig. 5:**
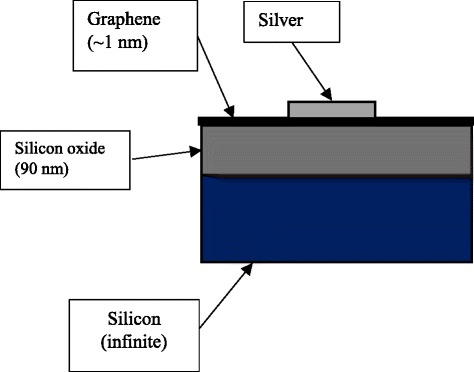
Side view of drawn model used in CST simulation

**Fig. 6:**
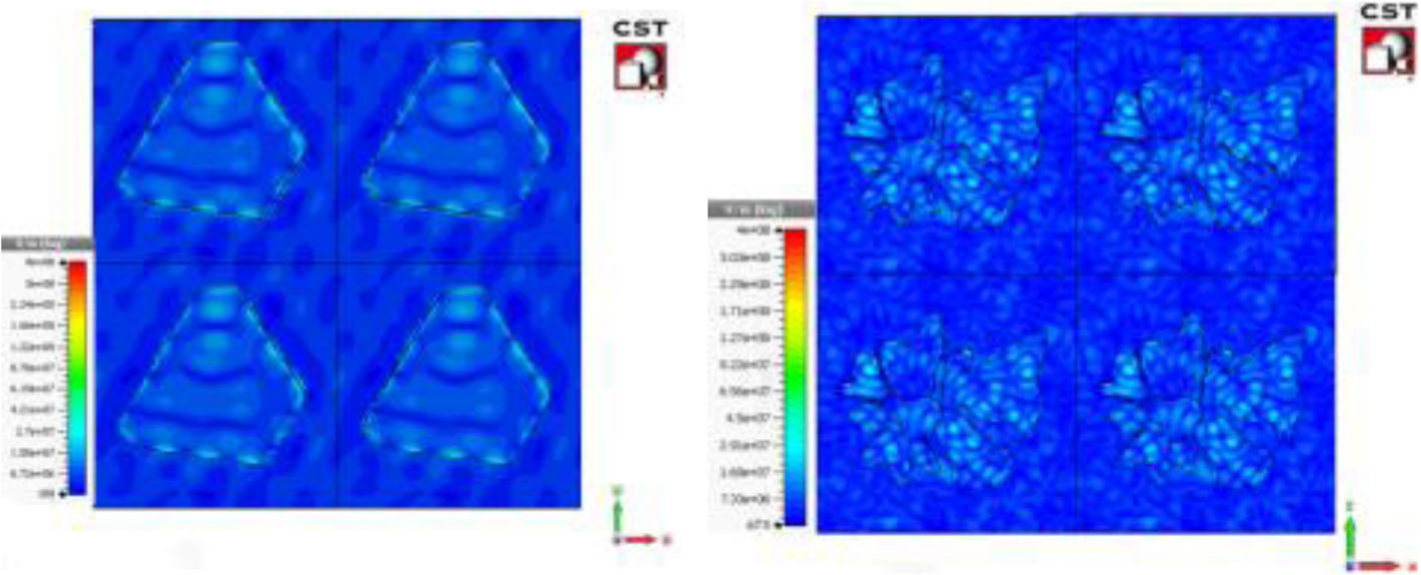
Electric field intensity distributions above the Au surface under a 425 nm incident wave polarized in the *y* direction

For the computer simulation, CST Microwave Studio was used to simulate the absorption characteristics of the samples, and a finite element method (FEM) was used when conducting this simulation. The structures of small-size Ag nanoparticles and the micro-flower shapes were drawn based on the obtained SEM images. The structures are shown in Fig. 4, and the dimensions for the nanoparticles were based on the data obtained from the surface profiler. To make the drawn model similar to the real sample, all of the Ag nanoparticles were drawn on the surface of graphene with silicon dioxide and silicon as a substrate, as shown in Fig. 5. In Fig. 6, the absorption spectrum of the Ag nanoparticles exhibited LSPR characteristics at 425 nm after a 5-min deposition time. This differed from 2.5 min, where a slight absorption peak was observed due to fewer silver atoms deposited on the graphene surfaces, which caused a low SPR effect to occur on the nanoparticles. The surface plasmon resonance of the metallic particles can be explained on the basis of the Mie theory, which concerns the dipolar oscillations of the free electrons in the conduction band that occupies energy states above the Fermi level [21]; these electrons do not oscillate at certain frequencies, causing depletion in the plasmon absorption band.

**Fig. 7:**
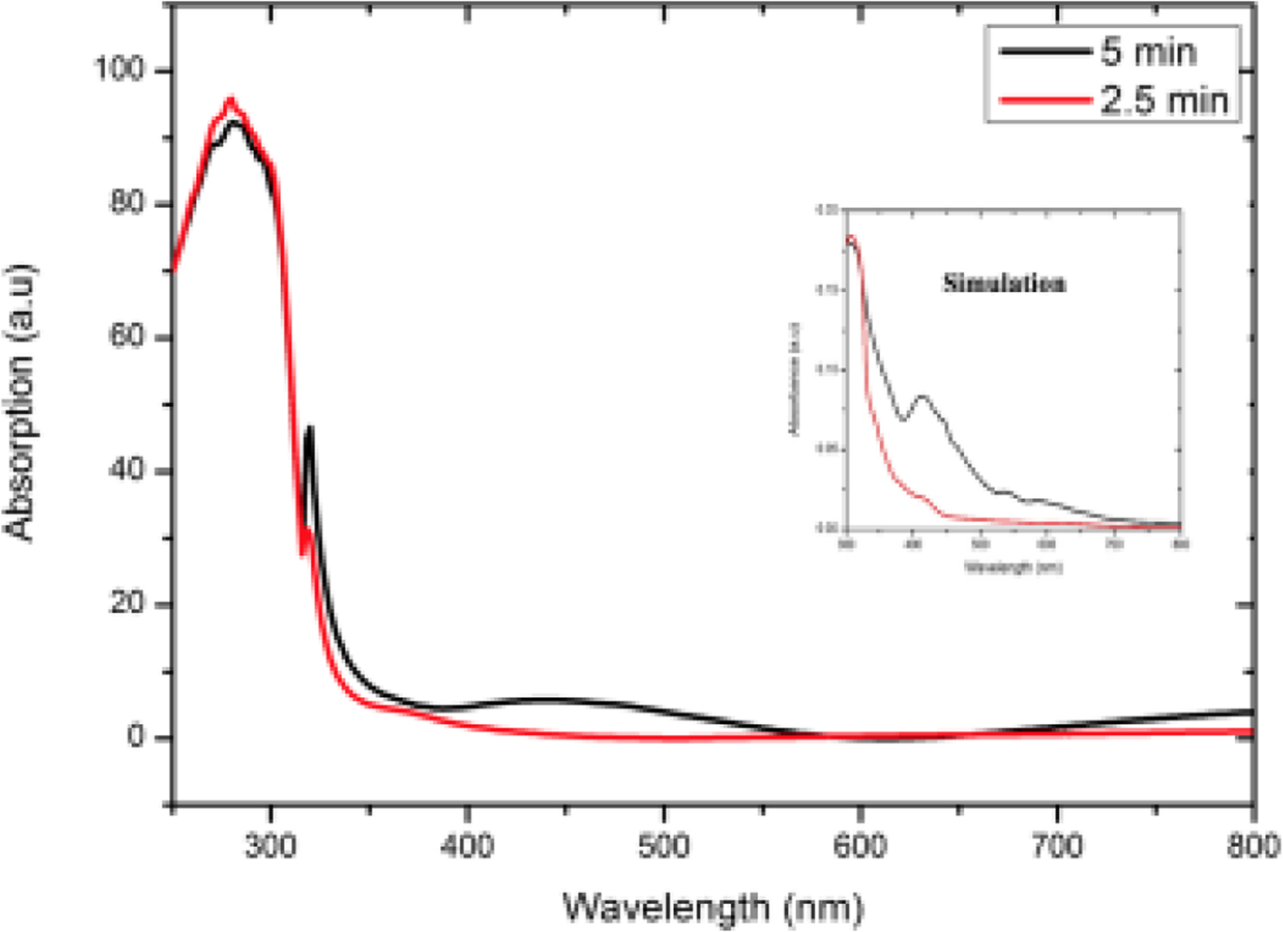
UV-vis absorption spectra for Ag flower-like structure on graphene substrate, and the *inset* shows simulated absorption spectrum at different deposition time

**Fig. 8:**
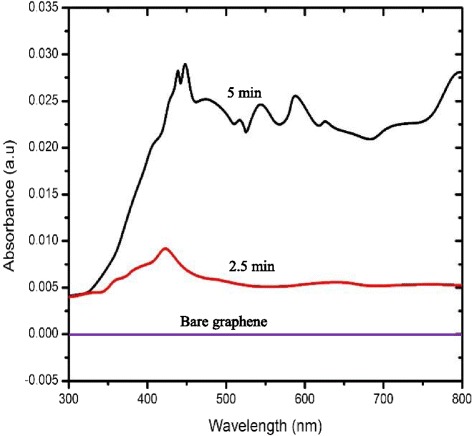
Simulated absorption spectrum of Ag nanoparticles coated on the graphene with different deposition time

The characterization of Ag nanoparticles was done in the simulation, and the light absorption spectrum was analyzed, as shown in Figs. 6, 7, and 8. The diffusion of Ag nanoparticles on the graphene surface was due to the SPR absorption contribution to the collective oscillation of electrons that was caused by an electromagnetic field [22]. On the other hand, EM is well accepted as the main mechanism of SERS for metallic nanostructures. The local electric field near a nanostructure is greatly enhanced by the LSPRs, which produce an enhancement that is highly localized to the metal surface. Figure 6 shows the distribution of the electric field at its highest intensity near 425 nm. In the simulation, the nanostructures are illuminated by a plane wave incident from the *z* direction normal to the surface, with the electric field polarized in the *y* direction.

Figure 7 shows a room temperature UV-visible absorption spectrum of the synthesized Ag structure, and here, the measurement is basically on the reflection of the sample. The maximum peak occurred at 280 nm in this measurement, and the spectrum shows no obvious change between both deposition times, possibly because of the location of the structures, as the inset graph shows the absorption spectrum from the simulation in which the structures are in constant arrangement and spacing. Figure 8 shows the simulated absorption spectra of Ag nanoparticles coated on the graphene surface with the deposition times of 2.5 and 5 min. It was observed that the enhancement occurred in the absorbance of the graphene after the deposition of Ag. The absorption spectrum exhibits the characteristics of SPR at 425 nm after 2.5 min, when the deposition time reached 5 min, and the absorption peak was redshifted to 450 nm. The redshifted wavelength and the increase in the absorption spectrum were caused by the increase in nanoparticle size [23]. Moreover, the enhancement of the absorbance peak occurred because of the SPR effects that occurred on the Ag micro-flower nanoparticles. In addition, a strong interaction between the Ag nanoparticles and graphene shows the alteration in the electronic and optical properties of graphene due to the presence of Ag nanoparticles. Several peaks were observed after the 5-min deposition time that represents the existence of a strong LSPR excitation on the surfaces of the non-uniform corners and edges of the Ag micro-flower structures. As mentioned earlier, the experimental results show that the intensity of the LSPR was remarkably enhanced after 2.5 and 5 min deposition times. The enhancement of the intensity occurred due to the increment of hotspots that was available on the graphene surfaces [24]. This is similar to simulation results that show an increase in the intensity of LSPR with deposition time.

## Conclusions

In this research, an electrochemical deposition technique with an optimum potential value of 0.6 V was used to deposit Ag nanoparticles on graphene surfaces. With a longer deposition processing time of 5 min, Ag micro-flower structures were formed, as many Ag nanoparticles stacked together in layers. It was also observed that when the deposition time increases, the distance between the micro-flower particles decreased, causing a drastic enhancement in Raman intensity. The enhancement in Raman intensity was also caused by the strong LSPR effect on the surface of the Ag nanoparticles. Similar to the simulation results, a redshift of the wavelength and an increment of the absorption peak of graphene can be observed due to the increase of Ag nanoparticle size. In addition, the increase of the absorption peak occurred because of the strong interaction between the Ag nanoparticles and the excitation light. Generally, this work can be further applied, ideally for terahertz applications and tunable detectors.

## Abbreviations

CA: Chronoamperometry
CV: Cyclic voltammetry
FEM: Finite element method
FESEM: Field-emission scanning electron microscope
LSPR: Localized surface plasmon resonance
NPs: Nanoparticles
SPR: Surface plasmon resonance
